# Inflammatory burden and persistent CT lung abnormalities in COVID-19 patients

**DOI:** 10.1038/s41598-022-08026-1

**Published:** 2022-03-11

**Authors:** Giulia Besutti, Paolo Giorgi Rossi, Marta Ottone, Lucia Spaggiari, Simone Canovi, Filippo Monelli, Efrem Bonelli, Tommaso Fasano, Nicola Sverzellati, Andrea Caruso, Nicola Facciolongo, Giulia Ghidoni, Anna Simonazzi, Mauro Iori, Andrea Nitrosi, Stefania Fugazzaro, Stefania Costi, Stefania Croci, Elisabetta Teopompi, Annalisa Gallina, Marco Massari, Giovanni Dolci, Fabio Sampaolesi, Pierpaolo Pattacini, Carlo Salvarani

**Affiliations:** 1 Radiology Unit, Department of Diagnostic Imaging and Laboratory Medicine, Azienda USL – IRCCS di Reggio Emilia, Viale Risorgimento 80, 42123 Reggio Emilia, Italy; 2 Department of Medical and Surgical Sciences, University of Modena and Reggio Emilia, Modena, Italy; 3 Epidemiology Unit, Azienda USL – IRCCS di Reggio Emilia, 42123 Reggio Emilia, Italy; 4 Clinical Chemistry and Endocrinology Laboratory, Azienda USL-IRCCS Di Reggio Emilia, 42123 Reggio Emilia, Italy; 5 Clinical and Experimental PhD Program, University of Reggio Emilia, 41124 Modena, Italy; 6 Radiology Unit, Department of Medicine and Surgery, University of Parma, 43126 Parma, Italy; 7 Rheumatology Unit, Azienda USL – IRCCS di Reggio Emilia, 42123 Reggio Emilia, Italy; 8 Respiratory Diseases Unit, Azienda USL – IRCCS di Reggio Emilia, 42123 Reggio Emilia, Italy; 9 Medical Physics Unit, Azienda USL – IRCCS di Reggio Emilia, 42123 Reggio Emilia, Italy; 10 Physical Medicine and Rehabilitation Unit, Azienda USL – IRCCS di Reggio Emilia, 42123 Reggio Emilia, Italy; 11 Scientific Directorate Azienda, USL - IRCCS Di Reggio Emilia, 42123 Reggio Emilia, Italy; 12 Department of Surgery, Medicine, Dentistry and Morphological Sciences With Interest in Transplant, Oncology and Regenerative Medicine, University of Modena and Reggio Emilia, 41124 Modena, Italy; 13 Clinical Immunology, Allergy and Advanced Biotechnologies Unit, Azienda USL – IRCCS di Reggio Emilia, 42123 Reggio Emilia, Italy; 14 Multidisciplinary Internal Medicine Unit, Guastalla Hospital, Azienda USL – IRCCS di Reggio Emilia, 42123 Reggio Emilia, Italy; 15 Infectious Diseases Unit, Azienda USL – IRCCS di Reggio Emilia, 42123 Reggio Emilia, Italy

**Keywords:** Medical research, Pathogenesis

## Abstract

Inflammatory burden is associated with COVID-19 severity and outcomes. Residual computed tomography (CT) lung abnormalities have been reported after COVID-19. The aim was to evaluate the association between inflammatory burden during COVID-19 and residual lung CT abnormalities collected on follow-up CT scans performed 2–3 and 6–7 months after COVID-19, in severe COVID-19 pneumonia survivors. C-reactive protein (CRP) curves describing inflammatory burden during the clinical course were built, and CRP peaks, velocities of increase, and integrals were calculated. Other putative determinants were age, sex, mechanical ventilation, lowest PaO2/FiO2 ratio, D-dimer peak, and length of hospital stay (LOS). Of the 259 included patients (median age 65 years; 30.5% females), 202 (78%) and 100 (38.6%) had residual, predominantly non-fibrotic, abnormalities at 2–3 and 6–7 months, respectively. In age- and sex-adjusted models, best CRP predictors for residual abnormalities were CRP peak (odds ratio [OR] for one standard deviation [SD] increase = 1.79; 95% confidence interval [CI] = 1.23–2.62) at 2–3 months and CRP integral (OR for one SD increase = 2.24; 95%CI = 1.53–3.28) at 6–7 months. Hence, inflammation is associated with short- and medium-term lung damage in COVID-19. Other severity measures, including mechanical ventilation and LOS, but not D-dimer, were mediators of the relationship between CRP and residual abnormalities.

## Introduction

Coronavirus Disease 2019 (COVID‐19) affected about 150 million subjects worldwide by the end of April 2021, with a case fatality rate ranging from 1.5% to 15% in different countries and phases of the pandemic^1,2^. Most severe COVID-19 patients develop acute respiratory distress syndrome with features of diffuse alveolar damage^3^.

Early studies on radiological follow-up of severe COVID-19 pneumonia showed residual abnormalities at follow-up CT scan, with a high prevalence especially in the short- and medium-term^4–8^. The clinical significance of these abnormal findings is unclear and the degree of persistency of residual pulmonary abnormalities will remain uncertain until more longer-term follow-up data are available^6–9^.

In a study including 114 survivors of severe COVID-19 with six-month follow-up CT scan, residual ground-glass opacities (GGO) or interstitial thickening, and CT fibrotic-like features (e.g., traction bronchiectasis, parenchymal bands, and/or honeycombing) were reported in 27%, and 35% of cases, respectively^5^. The presence of residual fibrotic-like abnormalities on CT was associated with older age, indices of higher disease severity, and mechanical ventilation^5^.

During the disease course, pulmonary and multiorgan damage is highly dependent on immune response and inflammation. In fact, inflammatory markers have been linked with poorer disease outcomes^10–12^. C-reactive protein (CRP) is the most widely used indicator of acute-phase response in clinical practice. CRP circulating concentrations increase rapidly and markedly in response to the onset of inflammation, and its levels decline with a half-life of approximately 20 h after resolution of the underlying inflammatory stimulus^13^. The rapid kinetics of circulating CRP explains why both CRP peak and the velocity of increase have been previously used in the definition of hyperinflammation in COVID-19 patients^12^.

The aim of this study was to evaluate the association between the degree of systemic inflammatory response during COVID-19 disease course and the presence of residual CT abnormalities at 2–3 and 6–7 months after COVID-19 pneumonia.

## Methods

### Setting

In the Reggio Emilia province (Northern Italy, 532,000 inhabitants) the first case of SARS-CoV-2 infection was diagnosed on 27 February 2020. The first pandemic wave lasted in Italy until May 2020. As of May 15, 2020, there had been 4863 RT-PCR-confirmed COVID-19 cases in the province. Details on hospital care in the province are reported in the Supplementary Materials.

### Study design and population

This was a single-centre retrospective cohort study based on routinely collected data, approved by the Area Vasta Emilia Nord (AVEN) Ethics Committee on 28 July 2020 (protocol number 855/2020/OSS/AUSLRE). The study was conducted in accordance with relevant guidelines and regulations, particularly with the Declaration of Helsinki. All consecutive COVID-19 patients who underwent a follow-up CT scan 2–3 months after COVID-19 pneumonia diagnosis were enrolled. In our institution, a routine 2–3 month follow-up CT scan was proposed to all COVID-19 survivors who had been hospitalised during the disease course for severe pneumonia with the following clinical-radiological features: respiratory failure during hospital stay (history of invasive or non-invasive mechanical ventilation and/or tocilizumab administration) or total extent of disease > 40% at baseline CT. A second CT scan at 6–7 months was proposed if residual CT abnormalities were present at 2–3 months. Given the retrospective nature of the cohort building, the need for informed consent was waived by the Area Vasta Emilia Nord Ethics Committee, after all reasonable efforts have been made to contact the patients to collect consent.

### Data collection

The COVID-19 Surveillance Registry, coordinated by the National Institute of Health and implemented in each Local Health Authority, was used to retrieve data on the dates of symptom onset, diagnosis, and hospitalisation. Patients’ medical records were reviewed to collect data on comorbidities, COVID-19 treatment, and mechanical ventilation (invasive and non-invasive). Blood tests at Emergency Department presentation, and all the available levels of CRP, D-dimer, and PaO2/FiO2 measured during hospitalization were retrieved from the laboratory information system. Detailed measurement methods are reported in the Supplementary Materials. The visually estimated extent of disease was retrieved from the structured reports of baseline CT scans^14^.

### Putative determinants

To study the relationship between inflammatory reaction and persistent lung abnormalities, three descriptors of CRP curves during disease course were considered: the peak (i.e., the maximum observed CRP value), the integral (i.e., the area under the curve of CRP levels), and the velocity of increase (i.e., the peak value divided by the number of days from symptom onset to the day on which the peak was registered). Details of the methods used to compute the CRP descriptors are reported in the Supplementary Materials and Supplementary Fig. 1. Other known determinants of COVID-19 prognosis and residual lung abnormalities were considered as potential confounding factors and included in the adjusted models: age, sex, and history of chronic pulmonary conditions preceding COVID-19 pneumonia (current or previous smoking history and/or concurrent respiratory diseases such as asthma, chronic obstructive pulmonary disease, and so on). Other indicators and markers of disease severity were considered as potential mediators of the inflammatory effect: mechanical ventilation, lowest PaO2/FiO2 ratio and D-dimer peak during the disease course, and length of hospital stay (LOS). Other known prognostic factors for COVID-19 which are intrinsically linked to the severity of inflammation as upstream causes or long-term effects of chronic inflammation (i.e., cardiovascular diseases, type 2 diabetes, and obesity) were not used to adjust the models because they probably lie on the same causal pathway linking inflammation to COVID-19 outcomes.

### Follow-up CT scan

Acquisition parameters for follow-up CT scans are reported in the Supplementary Materials. Two radiologists retrospectively reviewed follow-up CT scans at 2–3 and 6–7 months by consensus reading, and a gestalt assessment (i.e. global visual evaluation of the dominant CT pattern^15^), comparing serial CT scans was jointly performed by the two radiologists. Three main CT evolution patterns were systematically assessed for both short- and medium-term follow-up CT scans: no residual or trivial CT abnormalities (e.g., subtle GGO and reticulations occupying < 5% lung parenchyma); residual CT abnormalities suggestive of organizing pneumonia (OP) sequelae without overt fibrotic features (e.g., GGO, perilobular opacities, parenchymal bands, bronchial dilatation, with no predominant subpleural distribution); residual CT abnormalities with fibrotic features suggestive of non-specific or usual interstitial pneumonia (GGO and/or reticulation with traction bronchiectasis and/or honeycombing). Moreover, the presence of CT findings typical of post-ventilatory damage (cicatricial emphysema, parenchymal bands, and traction bronchiectasis in the anterior portions of the upper lobes) was collected. CT extension of residual abnormalities at 2–3 and 6–7 months was collected as the visually-estimated percentage of lung parenchyma involved by residual abnormalities, and categorized in 4 groups: < 20%, 20–39%, 40–59%, and ≥ 60%.

Early resolution was defined as no residual CT abnormality at 2–3 months, late resolution was considered for residual CT abnormalities (both non-fibrotic and fibrotic) at 2–3 months and no residual CT abnormalities at 6–7 months, while persistent CT abnormalities were defined when residual CT abnormalities (both non-fibrotic and fibrotic) were still visible at 6–7 months.

### Data analysis

We report continuous variables as median (interquartile range—IQR) and categorical variables as numbers and percentages. P-values are reported as continuous measures, and no significance threshold was set. Spearman’ correlations between the three descriptors of CRP are reported.

In regression analysis, CRP peak, the logarithmic form of CRP velocity of increase, CRP integral, PaO2/FiO2, D-dimer, and LOS were standardized. We built logistic models adjusted for age and sex to evaluate the association between CRP curve descriptors and the presence of CT lung abnormalities at 2–3 and 6–7 months. The performances of models with different descriptors were compared using Pseudo R^2^, Akaike information criterion (AIC), and area under ROC curve (AUROC), and the models with the best values of these three statistics were used to identify the CRP descriptors for subsequent analyses. We also present models adjusted by age, sex and including one by one the other COVID-19 severity indicators, i.e. mechanical ventilation and standardized D-dimer peak, lowest PaO2/FiO2, and LOS. Sensitivity analyses were also conducted on the selected models excluding patients with post-ventilatory lung CT abnormalities and patients with reported active smoking habit to exclude that the associations were only linked to these conditions. Linear regression models adjusted for age, sex only and for age, sex, and baseline CT extension, were used to test the association of the selected CRP descriptors and the extension of residual abnormalities at 2–3 and 6–7 months.

To evaluate the role of other measures of disease severity, we performed a formal mediation analysis^16,17^ using mechanical ventilation (invasive and non-invasive), and the standardized variables D-dimer peak, lowest PaO2/FiO2, and LOS as putative mediators of the CRP descriptors that were selected to be the best predictors of persistent lung abnormalities; models were always adjusted for age and sex. Details on mediation analysis are reported in the Supplementary Materials.

Given the different recommendations on mechanical ventilation in older patients, we tested for an interaction between age and PaO2/FiO2 or mechanical ventilation in their effect on outcomes.

Mediation analysis was performed using “medflex” package in R 3.4.0; the other analyses were conducted using Stata 13.0 SE (Stata Corporation, Texas, TX).

## Results

### Study population

**Figure 1:**
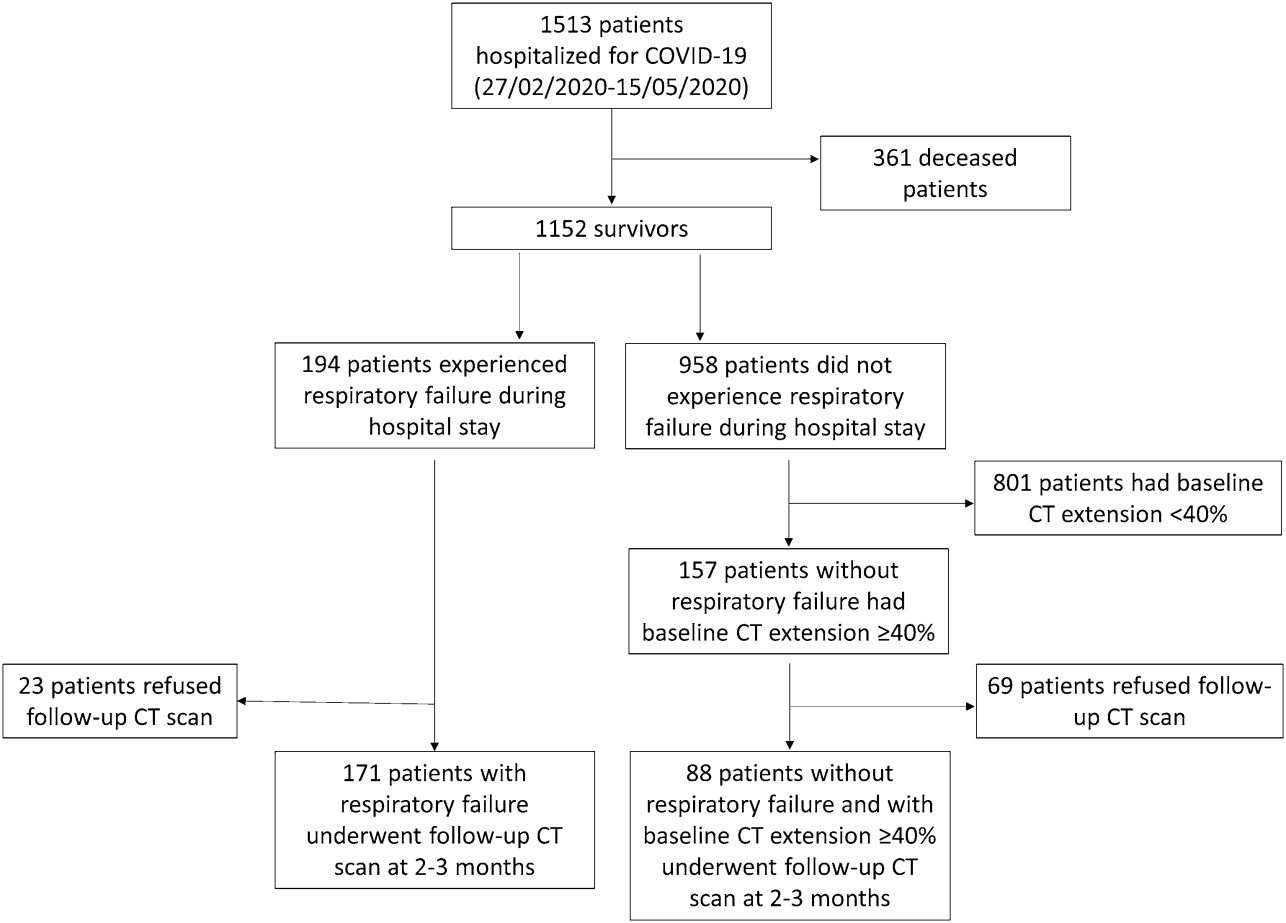
Flowchart representing patient enrolment.

During the first wave of the COVID-19 pandemic in the Reggio Emilia province (27 February – 15 May 2020), 1513 residents were hospitalised due to COVID-19. Of these, 361 patients died. Among the 1152 survivors, 194 patients experienced a respiratory failure during hospitalisation, and 157 other patients had a baseline total CT extent of ≥ 40% (Fig. 1). Of the patients who had an indication for follow-up CT scan, 259 patients (median age 65 years, 30.5% females) underwent a follow-up CT scan at 2–3 months and were included in the study; 241 of them had a second follow-up CT scan at 6–7 months.

Clinical characteristics of the included patients are reported in Table 1 and Supplementary Table 1. CRP was measured on average every 3 days (79 h), for a total average of 7.4 times during the clinical course of disease. All CRP descriptors were higher in patients with persistent abnormal findings (Table 1). The distribution of CRP curve descriptors in the whole population are described in Supplementary Fig. 2. CRP peak was strongly correlated with CRP velocity of increase and CRP integral, while the correlation between CRP integral and velocity of increase was weaker (Supplementary Table 2).

**Table 1:** Baseline putative determinants of CT lung abnormalities during follow-up in the whole cohort, and only in patients with CT lung abnormalities at 2–3 and at 6–7 months. IQR, interquartile range; COPD, chronic obstructive pulmonary disease; CRP, C-reactive protein; NIV, non-invasive ventilation; PaO2, arterial partial pressure of oxygen; FiO2, inspiratory fraction of oxygen. Mechanical ventilation refers to invasive and/or non-invasive mechanical ventilation. * Pearson’s chi-squared test or Fisher exact test and p-value for the hypothesis of independence in the two-way table; ** p-value for non-parametric equality-of-medians test; *** PaO2/FiO2 was not available (missing value) for 12 patients.

	All patients	CT lung abnormalities at 2–3 months		CT lung abnormalities at 6–7 months	
	N	N (%col)	P*	N (%col)	P*
	259	202 (78.0)		100 (38.6)	
Age (years), median (IQR)	65 (57–73)	67 (59–74)	< 0.001**	68 (61–75)	< 0.001**
Female sex, n (%)	79 (30.5)	60 (29.7)	0.599	31 (31.0)	0.973
Smoker, n (%)					
Never	223 (86.1)	170 (84.2)	0.120	83 (83.0)	0.569
Previous	32 (12.4)	29 (14.4)		15 (15.0)	
Current	4 (1.5)	3 (1.49)		2 (2.0)	
COPD, n (%)	9 (3.5)	8 (4.0)	0.688	5 (5.0)	0.282
Asthma, n (%)	9 (3.5)	7 (3.5)	1.000	4 (4.0)	1.000
Baseline CRP (mg/dl), median (IQR)	8.8 (4.4–15.6)	9.6 (4.9–15.8)	0.106**	11.9 (5.5–16.3)	0.001**
CRP peak (mg/dl), median (IQR)	15.6 (9.5–22.5)	16.2 (10.3–24.0)	0.012**	17.6 (12.8–28.1)	0.001**
CRP velocity (mg/dl/day), median (IQR)	1.2 (0.77–2.02)	1.3 (0.8–2.0)	0.309**	1.4 (0.9–2.4)	0.059**
CRP integral, median (IQR)	164 (87–256)	180 (105–287)	0.002**	198 (125–360)	< 0.001**
NIV, n (%)	102 (39.4)	94 (46.5)	< 0.001	52 (52.0)	0.002
Invasive ventilation, n (%)	30 (11.6)	27 (13.4)	0.105	21 (21.0)	0.001
Mechanical ventilation, n (%)	116 (44.8)	106 (52.5)	< 0.001	62 (62.0)	< 0.001
Lowest PaO2/FiO2 (mmHg), median (IQR)***	129 (85–237)	115 (82–205)	< 0.001	97 (79–168)	< 0.001
D-dimer peak (ng/ml), median (IQR)	1637 (832–4043)	1984 (952–4565)	0.003	2599 (1225–5254)	< 0.001
Length of hospital stay (days), median (IQR)	17 (11–29)	20 (14–30)	< 0.001	22 (16–40)	< 0.001

### Follow-up CT scan

**Figure 2:**
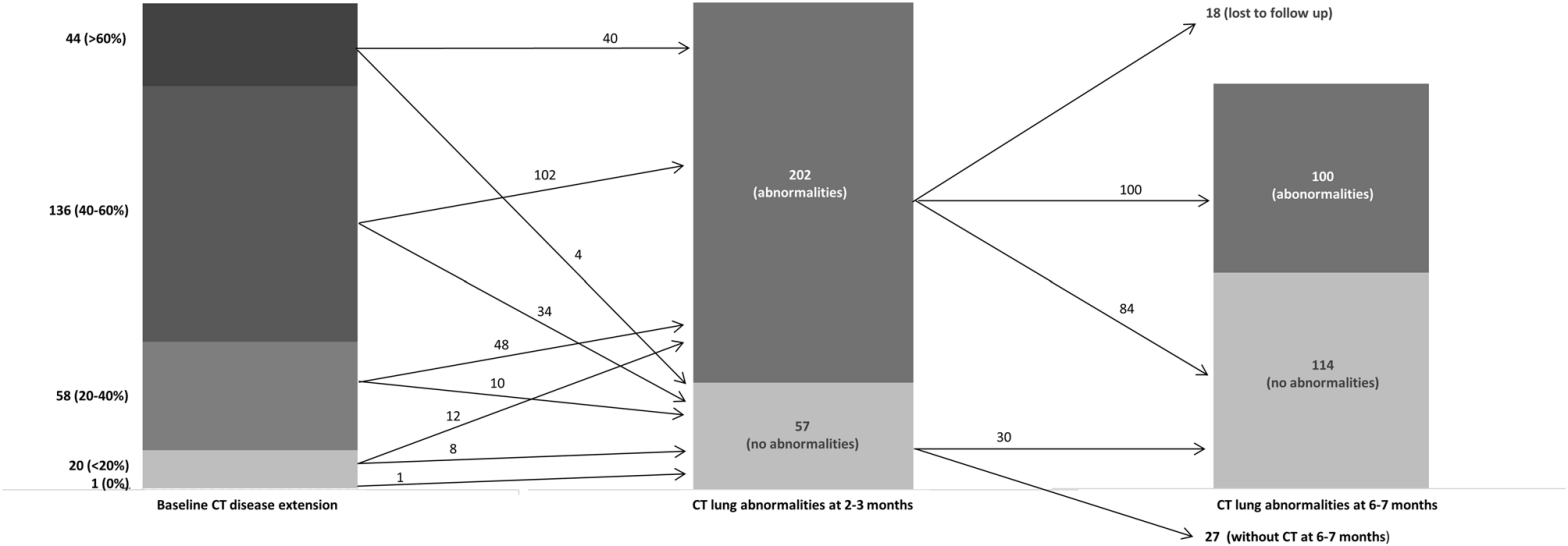
Graphic representation of patients’ follow-up, including the number of patients with different degrees of baseline parenchymal extension and patients with or without residual abnormalities at the two follow-up timepoints.

**Figure 3:**
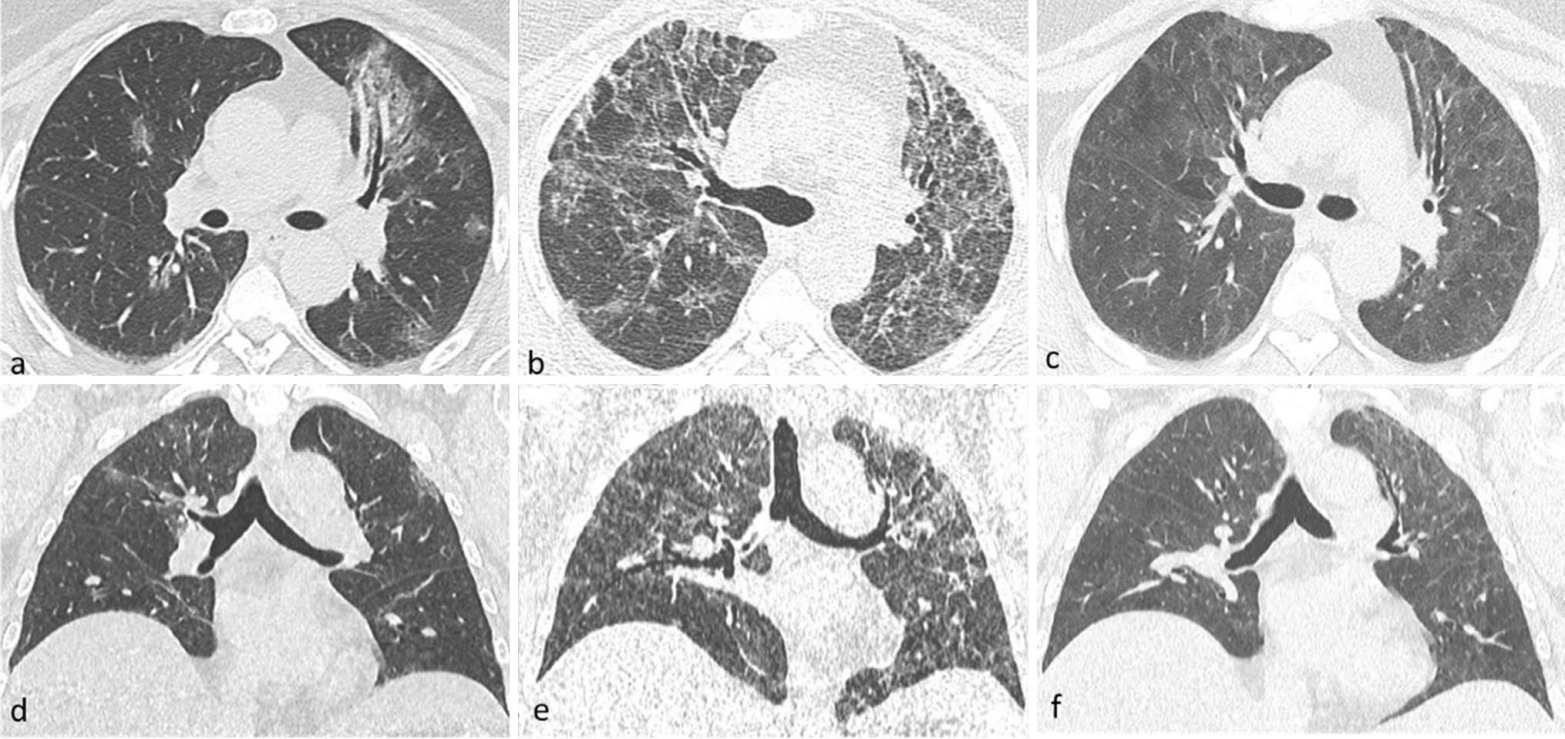
Disease evolution pattern consistent with OP sequelae. Axial (**a**–**c**) and coronal (**d**–**f**) CT images showing patchy ground glass opacities at baseline (**a**–**d**), diffuse ground-glass opacities with septal thickening, and perilobular opacities at 2–3 months (**b**–**e**), and subtle diffuse ground-glass with no CT features of lung fibrosis at 6–7 months (**c**–**f**).

**Figure 4:**
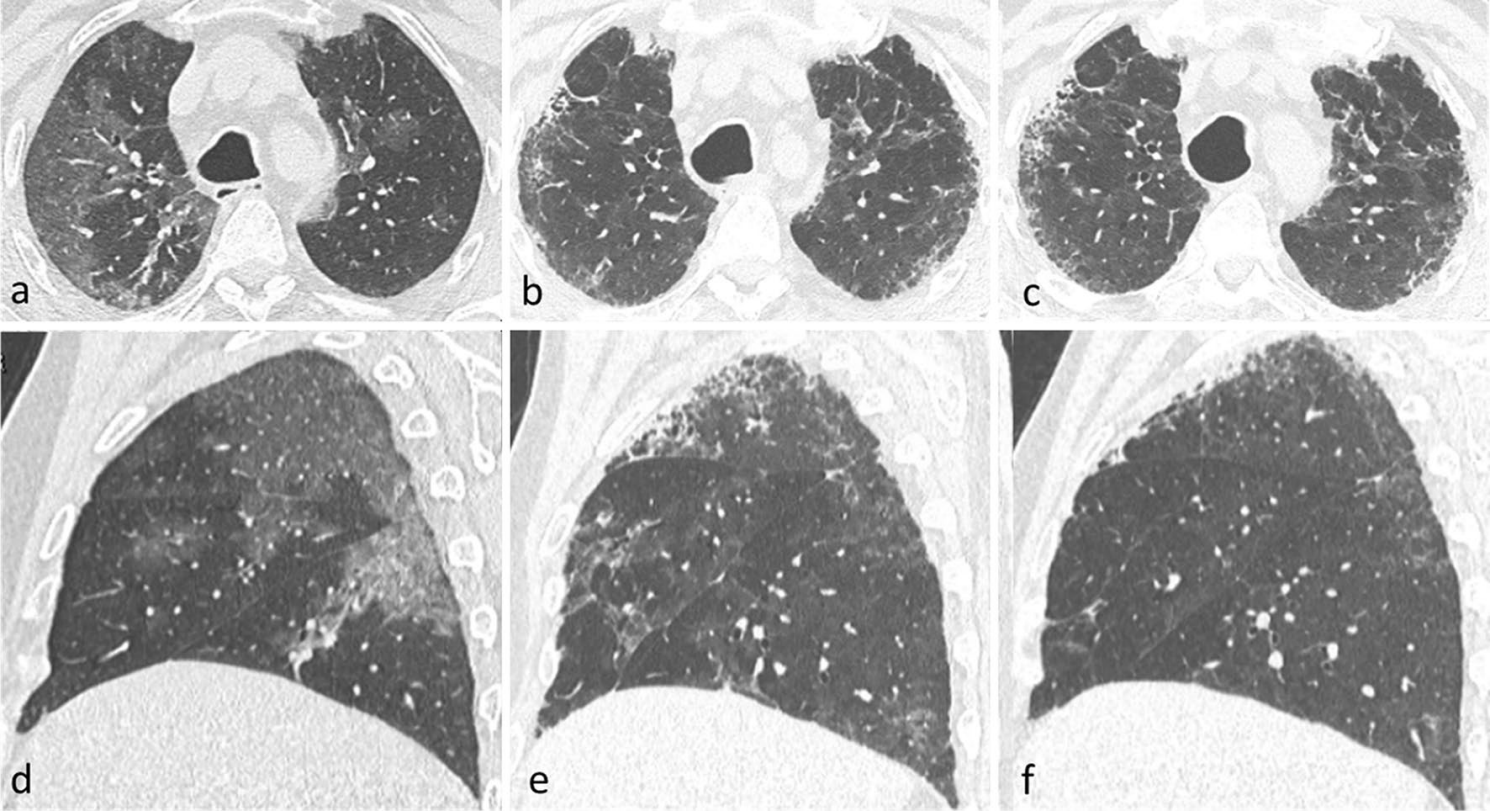
Disease evolution pattern consistent with fibrotic abnormalities (Non-Specific Interstitial Pneumonia, NSIP pattern). Axial (**a**–**c**) and sagittal (**d**–**f**) CT images showing patchy ground glass opacities at baseline (**a**–**d**), subtle residual ground-glass opacities, and appearance of subpleural reticulation and traction bronchiectasis in upper lobes at 2–3 months (**b**–**e**) and at 6–7 months (**c**–**f**).

**Figure 5:**
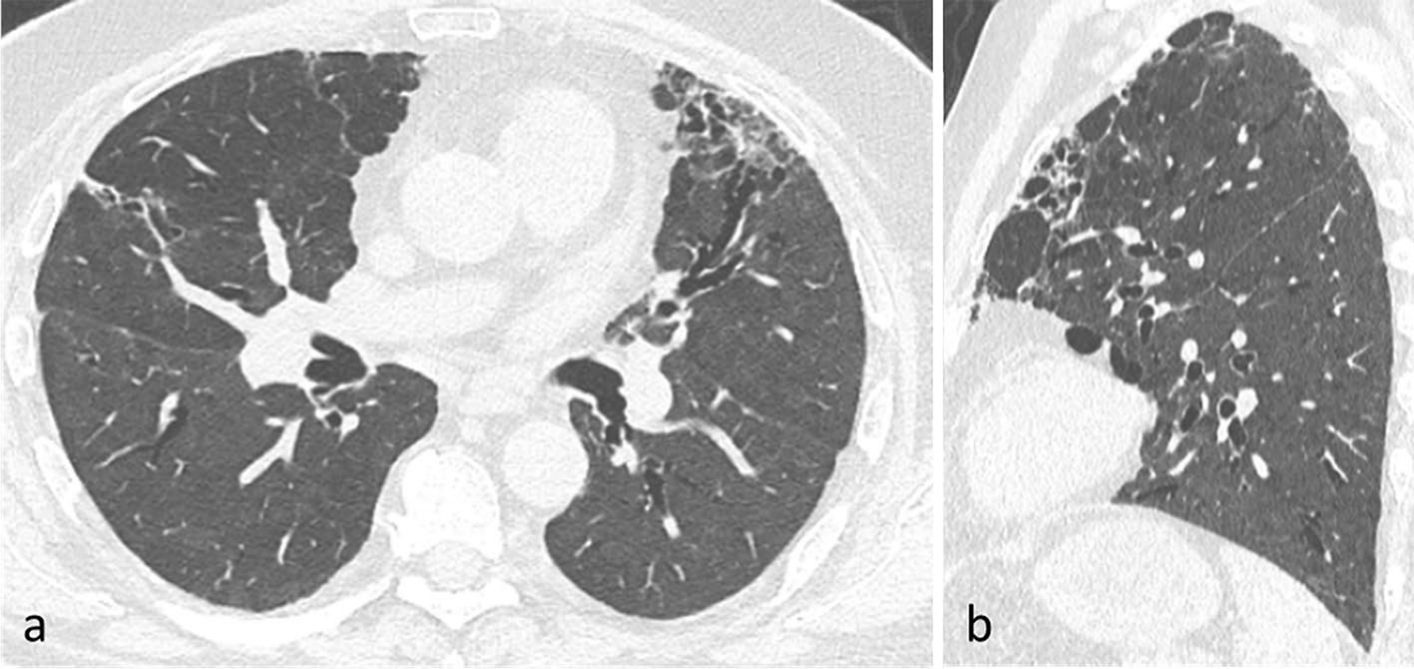
axial (**a**) and sagittal (**b**) CT images representing changes typical of post-ventilatory damage, characterized by cicatricial emphysema, parenchymal bands, and traction bronchiectasis in the anterior portions of upper lobes.

The first and second time point follow-up CT scans were performed at 74 ± 12 days, and at 191 ± 12 days from baseline CT scans, respectively. A total of 57/259 (22%) patients had an early resolution of lung damage, while 202/259 (78%) had non-trivial residual CT abnormalities at 2–3 months. Of these, 18/202 (8.9%) patients refused to undergo a second follow-up CT scan, 84 (41.6% of those retested, 32.4% of the initial sample) had no residual abnormalities at 6–7 months (late resolution), and 100 (49.5% of those retested, 38.6% of the initial sample) still had residual abnormalities at 6–7 months (Fig. 2). Most of the patients showed non-fibrotic CT abnormalities suggestive of OP sequelae (Fig. 3 and Supplementary Fig. 3). Only 9/100 patients showed fibrotic abnormalities at 6–7 months (Fig. 4), while 7 had CT findings typical of ventilation-induced damage (Fig. 5).

### CRP predictors of lung CT abnormalities at 2–3 and 6–7 months

By building different models adjusted for age and sex, we evaluated different combinations of standardized CRP curve descriptors in the prediction of persistent lung CT abnormalities. Other considered putative determinants, such as asthma or COPD and/or active smoking habit, were also tested as covariates, but they provided no added value to the models. The most accurate model for CT abnormalities at 2–3 months included CRP peak only (AUROC = 0.752, R^2^ = 0.138), while for residual abnormalities at 6–7 months the most accurate model included CRP integral only (AUROC = 0.748, R^2^ = 0.141) (Supplementary Table 3). In particular, a CRP peak increase of one SD was associated with an 80% increase in the risk of having residual abnormalities at 2–3 months (OR = 1.79; 95% CI = 1.23–2.62), while one SD increase in CRP integral was associated with a two-fold increase in the risk of having residual abnormalities at 6–7 months (OR = 2.24; 95% CI = 1.53–3.28) (Table 2). When excluding patients with ventilation-induced CT findings or active smokers, changes in ORs were negligible (Supplementary Table 4). In linear regression models, CRP peak and CRP integral were associated with CT extension of residual abnormalities at 2–3 and 6–7 months, respectively, both when adjusting for age and sex only and when adjusting also for baseline CT extension (Supplementary Table 5).

**Table 2:** Models for CT abnormalities at 2–3 and at 6–7 months including age, sex, and standardized CRP curve descriptors alone (Model 1) and adding other standardized measures of disease severity not directly (or not only) linked to hyperinflammation: mechanical ventilation (Model 2), lowest PaO2/FiO2 (Model 3), D-dimer peak (Model 4), length of hospital stay (Model 5). Std, standardized; OR, odds ratio; CI, confidence interval; AUC, area under the ROC curve; CRP, C-reactive protein. For standardized variables we report OR for one standard deviation increase of the variable; for age we report the OR for one year increase.

	CT lung abnormalities at 2–3 months				CT lung abnormalities at 6–7 months			
	N	OR (95% CI)	P	AUC	N	OR (95% CI)	P	AUC
**Model 1**	259			0.752	241			0.748
CRP peak std		1.79 (1.23–2.62)	0.003			–		
CRP integral std		–				2.24 (1.53–3.28)	< 0.001	
Age		1.07 (1.04–1.10)	< 0.001			1.06 (1.03–1.09)	< 0.001	
Sex M		1				1		
F		0.80 (0.40–1.59)	0.521			0.94 (0.51–1.75)	0.843	
**Model 2**	259			0.824	241			0.780
CRP peak std		1.37 (0.94–2.00)	0.099			–		
CRP integral std		–				1.86 (1.25–2.76)	0.002	
Age		1.09 (1.05–1.12)	< 0.001			1.07(1.04–1.10)	< 0.001	
Sex M		1				1		
F		0.73 (0.35–1.53)	0.400			0.88 (0.46–1.67)	0.692	
Mechanical Ventilation		7.13 (3.05–16.70)	< 0.001			3.32 (1.79–6.19)	< 0.001	
**Model 3**	247			0.791	229			0.746
CRP peak std		1.45 (0.97–2.18)	0.073			–		
CRP integral std		–				2.25 (1.50–3.39)	0.001	
Age		1.08 (1.05–1.11)	< 0.001			1.06 (1.04–1.09)	< 0.001	
Sex M		1				1		
F		0.83 (0.39–1.77)	0.630			0.93 (0.49–1.77)	0.834	
Lowest PaO2/FiO2 std		0.45 (0.26–0.77)	0.003			0.89 (0.67–1.20)	0.453	
**Model 4**	196			0.809	185			0.786
CRP peak std		1.82 (1.13–2.93)	0.013			–		
CRP integral std		–				2.03 (1.31–3.15)	0.002	
Age		1.08 (1.04–1.12)	< 0.001			1.07 (1.04–1.10)	< 0.001	
Sex M		1				1		
F		0.76(0.33–1.76)	0.517			0.80 (0.38–1.69)	0.566	
D-dimer peak std		1.68 (1.05–2.67)	0.029			1.70 (1.19–2.42)	0.003	
**Model 5**	259			0.797	241			0.769
CRP peak std		1.25 (0.84–1.88)	0.275			–		
CRP integral std		–				1.70 (1.12–2.59)	0.013	
Age		1.06 (1.03–1.09)	< 0.001			1.05 (1.03–1.08)	< 0.001	
Sex M		1				1		
F		0.74 (0.36–1.54)	0.421			0.89 (0.47–1.66)	0.705	
Length of hospital stay std		2.21 (1.42–3.43)	< 0.001			1.72 (1.18–2.51)	0.005	

### Other predictors of lung CT abnormalities at 2–3 and 6–7 months and mediation analyses

To test whether other indicators of disease severity not directly linked to hyperinflammation may have an independent role in predicting persistent lung CT abnormalities, we added other disease severity measures to the previously selected models (Table 2). An interaction between mechanical ventilation, PaO2/FiO2, and age in their effect on the outcomes was excluded. Only a slight increase in the models’ performance was observed when adding LOS or the lowest Pa/FiO2, while when adding mechanical ventilation, the AUC of the models improved more noticeably (from 0.752 to 0.824 for residual abnormalities at 2–3 months and from 0.748 to 0.780 for residual abnormalities at 6–7 months). Model performance was not directly comparable when adding D-dimer peak due to the presence of a remarkable number of missing values (n = 63). However, the increase in R^2^ suggests that D-dimer peak also adds a predictive value.

Adding other measures of disease severity partially reduced the association between CRP measures and the outcomes. This effect was absent or scarce for D-dimer and more apparent for the other measures of disease severity, except for the lowest PaO2/FiO2 in the model for 6–7-month abnormalities. The decrease in the association with the outcome is stronger for CRP peak when adding mechanical ventilation and for CRP integral when adding LOS.

**Figure 6:**
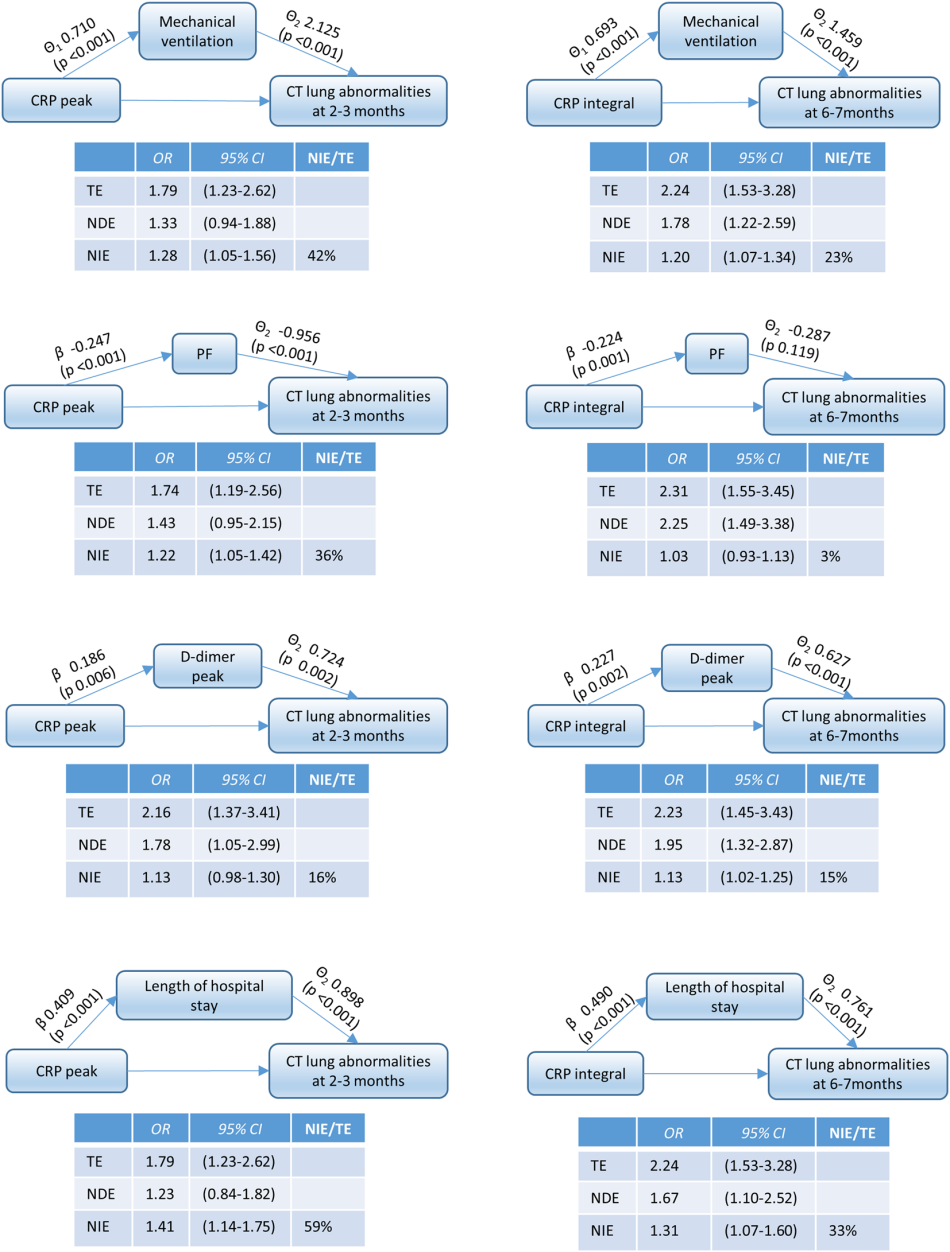
Path diagrams representing the mediation effect of mechanical ventilation, lowest PaO2/FiO2, D-dimer peak, and length of hospital stay in the relationship between CRP peak and CT lung abnormalities at 2–3 months and between CRP integral and CT lung abnormalities at 6–7 months. θ1 is the estimated effect (Ln OR) of CRP descriptors on mechanical ventilation adjusting for confounders. θ2 is the estimated effect (Ln OR) of mediators on persistent lung abnormalities adjusting for confounders. β is the correlation coefficient of CRP descriptors on mediators. Confounders include age and sex. In the tables we report the decomposition of the effect of CRP descriptors on persistent lung abnormalities. OR, odds ratio; TE, total effect; NDE, natural direct effect; NIE, natural indirect effect; NIE/TE, % mediation proportion.

The mediation analysis confirmed that mechanical ventilation and LOS were mediators of the effect of CRP descriptors on persistent CT abnormalities (Fig. 6). For example, 42% of the effect of CRP peak on short-term abnormalities and 23% of the effect of CRP integral on medium-term abnormalities was due to mechanical ventilation. A similar effect was found for the lowest PaO2/FiO2 ratio when considering abnormalities at 2–3 months. Instead, when considering abnormalities persisting at 6–7 months, PaO2/FiO2 was not associated with the outcome and therefore it could not be a mediator of CRP effect. Finally, the mediation effect of D-dimer peak on outcomes was negligible and could have been due to random fluctuations (the proportion of exposure effect due to the mediator on CT lung abnormalities at 2–3 months and at 6–7 months was 16% and 15%, respectively).

## Discussion

In a population of COVID-19 patients who were hospitalised for severe pneumonia, lung CT abnormalities were still visible in almost 80% patients after 2–3 months from disease onset, and in almost 40% after 6–7 months. CRP as a measure of systemic inflammatory response predicted residual CT abnormalities. The best predictors were CRP peak for residual abnormalities at 2–3 months and CRP integral for persistent abnormalities at 6–7 months. Other measures of disease severity, such as LOS, the lowest PaO2/FiO2, and the need for mechanical ventilation were mediators in the relationship between CRP and persistent CT lung abnormalities. While D-dimer was associated with persistent lung abnormalities, it was not a mediator of the inflammation effect.

Our results are consistent with prior follow-up studies, which reported late resolution or residual abnormalities at 6 months in up to 72% of subjects who had had COVID-19 pneumonia^4–7^. Most patients with residual CT abnormalities did not have overt CT features of lung fibrosis. Furthermore, more than 40% of patients who showed residual abnormalities at 2–3-month achieved complete resolution at 6–7 months. These findings corroborate the hypothesis that residual CT abnormalities are the expression of slow recovery in most cases, despite data on longer-term follow-up being needed to confirm that persistent fibrotic changes are not common. In fact, non-fibrotic abnormalities could be the expression of immature fibrosis (i.e. “fibroblastic changes” due to diffuse alveolar damage) remodelling^9^.

In the few available studies on the topic, several measures of disease severity predicted the presence of persistent lung abnormalities in COVID-19 patients^4,5^, but the role of inflammatory burden throughout the disease course has never been investigated.

Baseline CRP has been widely studied as a predictor of COVID-19 severity in the acute phase of the disease^10–12^. Some studies have also evaluated CRP trajectories during hospitalisation, showing that rapidly rising CRP levels may predict respiratory failure and that CRP peak is higher in patients who require intubation or die^12–18^. We show that persistent lung abnormalities are also linked to inflammatory burden, with most important predictors being CRP peak for persistent damage in a subacute phase (2–3 months), and CRP integral, reflecting both the intensity and the duration of the inflammatory reaction, for persistent abnormalities at 6–7 months.

Given the known association between CRP and lung damage in the acute phase^19^, it is not surprising that much of the effect of inflammatory burden on persistent lung abnormalities is mediated by proxies of lung failure severity, such as the need for mechanical ventilation and, at least for residual abnormalities at 2–3 months, the lowest PaO2/FiO2 as a measure of hypoxemia. The association between mechanical ventilation and other disease severity measures, including CRP, should be considered cautiously in our cohort, since the presence of mechanical ventilation was one of the sufficient, but not necessary inclusion criteria. Surprisingly, we did not observe an association between the lowest PaO2/FiO2 and 6–7 month abnormalities, therefore we did not find any mediation effect for this measure of disease severity.

Higher D-dimer levels have been widely associated with COVID-19 severity and mortality^20–22^, as the infection induces a coagulopathy with secondary hyperfibrinolysis in severe cases^23^. In our study, D-dimer peak was associated with persistent lung abnormalities, adding predictive value to the models without subtracting from the effect of CRP. Accordingly, its role as a mediator of the effect of inflammation on persistent abnormalities was modest, if any. Apparently, even if inflammation and coagulopathy are linked in COVID-19 patients^24–26^, CRP and D-dimer effects on persistent lung damage are independent, acting through different causal pathways.

This study has some limitations. First, only COVID-19 survivors who experienced severe pneumonia (extension > 40% at hospital admission and/or respiratory failure during hospitalisation) were routinely evaluated with follow-up CT scans and included in the study. Consequently, selection, survivorship and colliding bias cannot be excluded, and some of the observed associations between the severity measures themselves and between severity measures and the outcomes may be under or over-estimated. Mediation analysis should reduce the impact of colliding bias; while it must be clear that our findings are only meaningful for survivors of COVID-19 pneumonia identified as severe by baseline imaging or respiratory failure occurrence. Second, CRP curves were not always complete, and extrapolations were done. Many values were missing for D-dimer. CRP curve may be influenced by factors occurring during the disease course, such as bacterial superinfection, anti-inflammatory drugs, and post-ventilatory lung damage, but also by pre-existing pathological conditions. In our study, we can only observe the association between the inflammatory burden during COVID-19 and residual lung abnormalities, whatever was the mechanism underlying the inflammation, i.e. the direct response to the virus or rather an inflammatory response whose intensity can be modulated by therapies or by other pre-existing conditions. In fact, these factors cannot be considered confounders of our association, but factors probably lying on the same causal pathway, that starts from the existence of prognostic factors favoring an intense inflammatory response when SARS-CoV-2 infection occurs, and then includes the immune response following infection and the therapies we administered in response to infection and disease progression. Furthermore, we had no information on other potential confounders, such as pre-existing interstitial lung abnormalities. Nevertheless, our model building strategy was guided by the attempt to describe causal pathways, and our data suggest that therapy-linked variables, i.e. mechanical ventilation and LOS, are on the same causal pathway linking inflammation to persistent lung abnormalities. Our findings suggest that future studies trying to understand the etiopathogenesis of COVID-19 should carefully consider which variables should be placed in multivariable analyses. Otherwise, the risk is of hiding the causal links with upstream determinants, such as hyperinflammation markers, and of introducing effect mediators. Moreover, our study focused on persistent morphological abnormalities evaluated by means of CT scan, but the evaluation of the impact of residual CT abnormalities on symptoms and lung function tests is ongoing and is beyond the scope of the present study. Finally, our attempt to classify the pattern of CT abnormalities may be questionable. In fact, the non-fibrotic CT pattern may include features (e.g., GGO), that require more time to become overtly fibrotic on CT^9^. Also, the use of quantitative extension of residual changes does not necessarily improve the evaluation of CT findings evolution, which is variable and may include diffuse but very mild abnormalities (e.g. subtle GGO). However, the association of CRP with residual abnormalities was confirmed also by using CT visual extension as outcome measure.

In conclusion, higher inflammatory burden is associated with short- and medium-term lung damage. The peak value of CRP is the most important determinant of damage at 2–3 months, while both the duration and intensity of the inflammatory response become important for more long-lasting damage (6–7 months). Other measures of disease severity seem to be mediators of the inflammatory effect, except for D-dimer peak, confirming that the two pathways of inflammation and coagulopathy are partially independent, although both contribute to lung damage.

## Supplementary Information

Supplementary Information.

## Data Availability

Participant data that underlie the results reported in this manuscript will be shared after de-identification, beginning 6 months and ending at least 7 years after article publication, to researchers who provide a methodologically sound proposal with objectives consistent with those of the original study. Proposals and data access requests should be directed to the Area Vasta Emilia Nord (AVEN) Ethics Committee at CEReggioemilia@ausl.re.it as well as to the Authors at the Epidemiology Unit of AUSL–IRCCS di Reggio Emilia at info.epi@ausl.re.it, who are the data guardians. To gain access, data requestors will need to sign a data access agreement.

## Abbreviations

AIC: Akaike information criterion
AUROC: Area under ROC curve
COPD: Chronic obstructive pulmonary disease
CRP: C-reactive protein
CT: Computed Tomography
FiO2: Inspiratory fraction of oxygen
GGO: Ground glass opacities
IQR: Interquartile range
LOS: Lenght of hospital stay
NIV: Non-invasive ventilation
OP: Organizing Pneumonia
PaO2: Arterial partial pressure of oxygen

## References

[1] WHO Coronavirus Disease (COVID-19) Dashboard. [accessed on June 25, 2021]. Available at https://covid19.who.int

[2] Dong E, Du H, Gardner L (2020). An interactive web-based dashboard to track COVID-19 in real time. Lancet Infect Dis..

[3] Yang X (2020). Clinical course and outcomes of critically ill patients with SARS-CoV-2 pneumonia in Wuhan, China: a single-centered, retrospective, observational study. Lancet Respir. Med..

[4] So M, Kabata H, Fukunaga K, Takagi H, Kuno T (2021). Radiological and functional lung sequelae of COVID-19: a systematic review and meta-analysis. BMC Pulm. Med..

[5] Han X (2021). Six-month Follow-up Chest CT Findings after Severe COVID-19 Pneumonia. Radiology.

[6] Wu X (2021). 3-month, 6-month, 9-month, and 12-month respiratory outcomes in patients following COVID-19-related hospitalisation: a prospective study. Lancet Respir. Med..

[7] Caruso D (2021). Postacute Sequelae of COVID-19 Pneumonia: 6-month Chest CT Follow-up. Radiology.

[8] Zhang S (2021). Eight months follow-up study on pulmonary function, lung radiographic, and related physiological characteristics in COVID-19 survivors. Sci. Rep..

[9] Wells AU, Devaraj A, Desai SR (2021). Interstitial lung disease after COVID-19 infection: a catalog of uncertainties. Radiology.

[10] Hariyanto TI (2021). Inflammatory and hematologic markers as predictors of severe outcomes in COVID-19 infection: A systematic review and meta-analysis. Am. J. Emerg. Med..

[11] Akbari H (2020). The role of cytokine profile and lymphocyte subsets in the severity of coronavirus disease 2019 (COVID-19): a systematic review and meta-analysis. Life Sci..

[12] Manson JJ (2020). COVID-19-associated hyperinflammation and escalation of patient care: a retrospective longitudinal cohort study. Lancet Rheumatol..

[13] Rajab IM, Hart PC, Potempa LA (2020). How C-reactive protein structural isoforms with distinctive bioactivities affect disease progression. Front. Immunol..

[14] Besutti G (2020). Accuracy of CT in a cohort of symptomatic patients with suspected COVID-19 pneumonia during the outbreak peak in Italy. Eur. Radiol..

[15] Koontz NA, Gunderman RB (2008). Gestalt theory: implications for radiology education. AJR Am. J. Roentgenol..

[16] Vanderweele TJ, Vansteelandt S (2010). Odds ratios for mediation analysis for a dichotomous outcome. Am. J. Epidemiol..

[17] Wang W, Zhang B (2016). Assessing natural direct and indirect effects for a continuous exposure and a dichotomous outcome. J. Stat. Theory Pract..

[18] Mueller AA (2020). Inflammatory biomarker trends predict respiratory decline in COVID-19 patients. Cell Rep. Med..

[19] Canovi S (2021). The association between clinical laboratory data and chest CT findings explains disease severity in a large Italian cohort of COVID-19 patients. BMC Infect. Dis..

[20] Lippi G, Favaloro EJ (2020). D-dimer is associated with severity of coronavirus disease 2019: a pooled analysis. Thromb Haemost..

[21] Tang N, Li D, Wang X, Sun Z (2020). Abnormal coagulation parameters are associated with poor prognosis in patients with novel coronavirus pneumonia. J Thromb. Haemost..

[22] Perico L (2021). Immunity, endothelial injury and complement-induced coagulopathy in COVID-19. Nat. Rev. Nephrol..

[23] Iba T, Levy JH, Levi M, Connors JM, Thachil J (2020). Coagulopathy of coronavirus disease 2019. Crit. Care Med..

[24] Shorr AF, Thomas SJ, Alkins SA, Fitzpatrick TM, Ling GS (2002). D-dimer correlateswith proinflammatory cytokine levels and outcomes in critically ill patients. Chest.

[25] Xu Z (2020). Pathological findings of COVID-19 associated with acute respiratory distress syndrome. Lancet Respir. Med..

[26] Channappanavar R, Perlman S (2017). Pathogenic human coronavirus infections: causes and consequences of cytokine storm and immunopathology. Semin. Immunopathol..

