# Supplementary material for: Inflammatory burden and persistent CT lung abnormalities in COVID-19 patients
Source: Sci Rep. 2022 Mar 11;12:4270. doi: 10.1038/s41598-022-08026-1 (PMC8914439; doi:10.1038/s41598-022-08026-1)
Supplement: Supplementary file 1 — Supplementary Information. [file 41598_2022_8026_MOESM1_ESM.pdf]

Inflammatory burden and persistent CT lung abnormalities in COVID-19 patients

Giulia Besutti[1,2]*, Paolo Giorgi Rossi[3], Marta Ottone[3], Lucia Spaggiari[1], Simone Canovi[4], Filippo Monelli[1,2], Efrem Bonelli[1], Tommaso Fasano[4], Nicola Sverzellati[5], Andrea Caruso[6], Nicola Facciolongo[7], Giulia Ghidoni[7], Anna Simonazzi[7], Mauro Iori[8], Andrea Nitrosi[8], Stefania Fugazzaro[9], Stefania Costi[10], Stefania Croci[11], Elisabetta Teopompi[12], Annalisa Gallina[1], Marco Massari[13], Giovanni Dolci[13], Fabio Sampaolesi[13], Pierpaolo Pattacini[1], Carlo Salvarani[6].

1. Radiology Unit, Department of Diagnostic Imaging and Laboratory Medicine, Azienda USL – IRCCS di Reggio Emilia, 42123, Reggio Emilia, Italy.

2. Clinical and Experimental PhD program, University of Reggio Emilia, 41124, Modena, Italy.

3. Epidemiology Unit. Azienda USL – IRCCS di Reggio Emilia, 42123, Reggio Emilia, Italy.

4. Clinical Chemistry and Endocrinology Laboratory. Azienda USL-IRCCS di Reggio Emilia, 42123, Reggio Emilia, Italy

5. Radiology Unit, Department of Medicine and Surgery, University of Parma. 43126, Parma, Italy.

6. Rheumatology Unit, Azienda USL – IRCCS di Reggio Emilia, 42123, Reggio Emilia, Italy.

7. Respiratory Diseases Unit. Azienda USL – IRCCS di Reggio Emilia, 42123, Reggio Emilia, Italy.

8. Medical Physics Unit, Azienda USL – IRCCS di Reggio Emilia, 42123, Reggio Emilia, Italy.

9. Physical Medicine and Rehabilitation Unit, Azienda USL – IRCCS di Reggio Emilia, 42123, Reggio Emilia, Italy.

10. Scientific Directorate Azienda USL - IRCCS di Reggio Emilia, 42123, Reggio Emilia, Italy. Department of Surgery, Medicine, Dentistry and Morphological Sciences with Interest in Transplant, Oncology and Regenerative Medicine, University of Modena and Reggio Emilia, 41124, Modena, Italy.

11. Clinical Immunology, Allergy and Advanced Biotechnologies Unit, Azienda USL – IRCCS di Reggio Emilia, 42123, Reggio Emilia, Italy.

12. Multidisciplinary Internal Medicine Unit. Guastalla Hospital, Azienda USL – IRCCS di Reggio Emilia, 42123, Reggio Emilia, Italy.

13. Infectious Diseases Unit. Azienda USL – IRCCS di Reggio Emilia, 42123, Reggio Emilia, Italy.

Supplementary Material - Methods

Setting

In the Reggio Emilia province (Northern Italy, 532,000 inhabitants), Local Health Authority (LHA) hospital care is provided by six hospitals, with five emergency departments (ED) and one imaging department with centralized image reading. During the first pandemic wave, which lasted in Italy until May 2020, patients in the Reggio Emilia province with severe COVID-19 pneumonia were hospitalized primarily in the two main hospitals.

Methods for CRP and D-dimer measurement

CRP was measured in serum or plasma using the automated Siemens Immunoturbidimetric "wide range CRP" method on ADVIA-1800 analyzers (Siemenes Healthineers, Erlangen, Germany); D-dimer concentrations were measured on citrated plasma samples with Siemens "Innovance D-dimer" (reported in fibrinogen equivalent units) on Sysmex CS-5100 coagulometers (Sysmex Corporation, Kobe, Japan).

Methods for computing CRP descriptors

CRP peak: the highest value in (mg/dl) registered during the disease course

CRP integral: the area under the polygon designed by the linear interpolation between measured points in the graph with days from symptom onset and CRP mg/dl observed during the disease course (expressed as mg/dl*day). Computationally it is the $\sum_{1}^{n} CRPn$, where n is the day of disease from symptom onset and the CRP_n is the value of CRP registered or interpolated on the nth day.

If more than one CRP value was registered in one day, the average was considered. If for one or more days there was no CRP measured, the value was estimated by linear interpolation using the closest known values; for the days between symptom onset and the first measurement, a triangle was estimated interpolating the values from symptom onset and the first value assuming CRP=0 at day one of symptom onset; the course of inflammation ended when CRP reached a value of <1 mg/dl. For patients having the last measurement (usually at discharge) higher than 3 mg/dl we estimated the right tail of the curve using the average descending slope of the observed complete curves (ie, those with last observed value <1 mg/dl) with a similar CRP value (+/- 1) at the same day from symptom onset (or at the closest time). Curves ending with a value between 1 and 3 mg/dl were not completed through an estimate of the right tail. Indeed, the change in total values would be negligible, increasing the percentage of estimated data.

CRP velocity: the peak value divided by the number of days from symptom onset to the day of peak value (mg/dl/days).

For example, in Supplementary figure 1 we report a case of an estimated right tail (A) obtained by adding the observed time to CRP decrease in a complete curve (B). The shaded area below the drawn line is the estimated area.

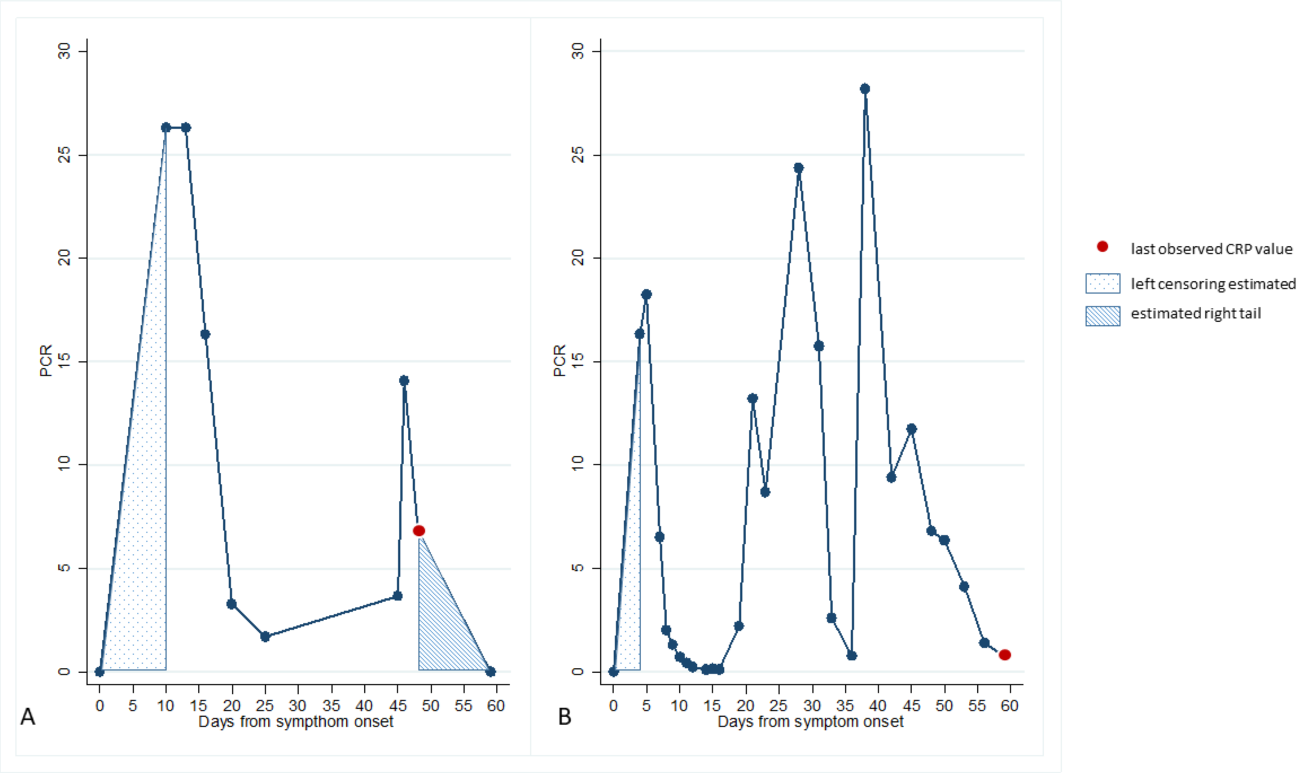

Supplementary Figure 1: Example of an estimated right tail (A) obtained by adding the observed time to CRP decrease in a complete curve (B). The shaded area below the drawn line is the estimated area.

Methods for follow-up CT scan acquisition parameters

Follow-up CT scans were performed using a 128-slice scanner (Somatom Definition Edge, Siemens Healthineers), without contrast media injection, with the patient in supine position during end-inspiration. Scanning parameters were tube voltage 120 KV, automatic tube current modulation, collimation width 0.625 or 1.25 mm, acquisition slice thickness 2.5 mm, and interval 1.25 mm. Images were reconstructed with a high-resolution algorithm at slice thickness 1.0/1.25 mm.

Methods for mediation analysis

Using the method provided by VanderWeele (13), we fitted natural effect models to estimate the natural direct (NDE) and natural indirect (NIE) effects of CRP peak on CT abnormalities at 2-3 months and CRP integral at 6-7 months on odds ratio (OR) scales. We also report the proportion of the mediation effect of each measure of disease severity estimated using logarithm of OR (14).

$$P(x)_{OR} = log(NIE(x)_{OR})/\log(TE_{OR})$$

where TE is the total effect.

Supplementary Table 1. Patients' pre-existing clinical conditions, as well as baseline laboratory and CT characteristics at the first hospital admission for COVID-19.

		Median (IQR)	min	max	Missing (n)
		N=259			
Comorbidities, n (%)					
	yes	207 (79.9)			
	no	52 (20.1)			
Hypertension, n (%)		152 (58.7)			
Diabetes, n (%)		55 (21.2)			
Cancer, n (%)		27 (10.4)			
Overweight/obesity, n (%)		59 (22.8)			
Chronic kidney failure, n (%)		9 (3.5)			
Liver diseases, n (%)		6 (2.3)			
Dyslipidemia, n (%)		51 (19.7)			
Asthma, n (%)		9 (3.5)			
Arrythmias, n (%)		13 (5.0)			
COPD, n (%)		9 (3.5)			
Cerebrovascular diseases, n (%)		12 (4.6)			
Cardiovascular diseases, n (%)		43 (16.6)			
Peripheral vascular diseases, n (%)		18 (7.0)			
Other, n (%)		33 (12.7)			
Baseline CT parenchymal extension, n (%)					
	<20%	21 (8.1)			
	20-39%	58 (22.4)			
	40-59%	136 (52.5)			
	≥60%	44 (17.0)			
CT parenchymal extension 2-3 months, n (%)					1
	<20%	129 (50.0)			
	20-39%	85 (33.0)			
	40-59%	35 (13.6)			
	≥60%	9 (3.5)			
CT parenchymal extension 6-7 months, n (%)					41
	<20%	151 (69.3)			
	20-39%	49 (22.5)			
	40-59%	15 (6.9)			
	≥60%	3 (1.4)			
CRP (mg/dL)		8.80 (4.40-15.56)	0.03	36.3	26
pH		7.46 (7.44-7.49)	3.40	7.61	40
PaO2 (mmHg)		64.8 (57.4-73.4)	27	709	34
PaCO2 (mmHg)		34.2 (31.4-37.1)	23.4	93.2	35
PaO2/FiO2 (mmHg)		291 (233-321)	31	681	63
White blood cell count ($*10^9$/L)		5.9 (4.5-7.8)	1.5	18.5	6
Neutrophil count ($*10^9$/L)		4.3 (3.1-6.3)	0.6	983.5	29
Lymphocyte count ($*10^9$/L)		0.94 (0.67-1.24)	0.13	55	29
D-dimer (ng/mL)		770 (457-1523)	171	35000	168
LDH (U/L)		580 (392-734)	105	1585	61

IQR, interquartile range; COPD, chronic obstructive pulmonary disease; CT, computed tomography; CRP, C-reactive protein; PaO2, arterial partial pressure of oxygen; PaCO2, arterial partial pressure of carbon dioxide; FIO2, inspiratory fraction of oxygen; LDH, lactate dehydrogenase.

Supplementary Figure 2

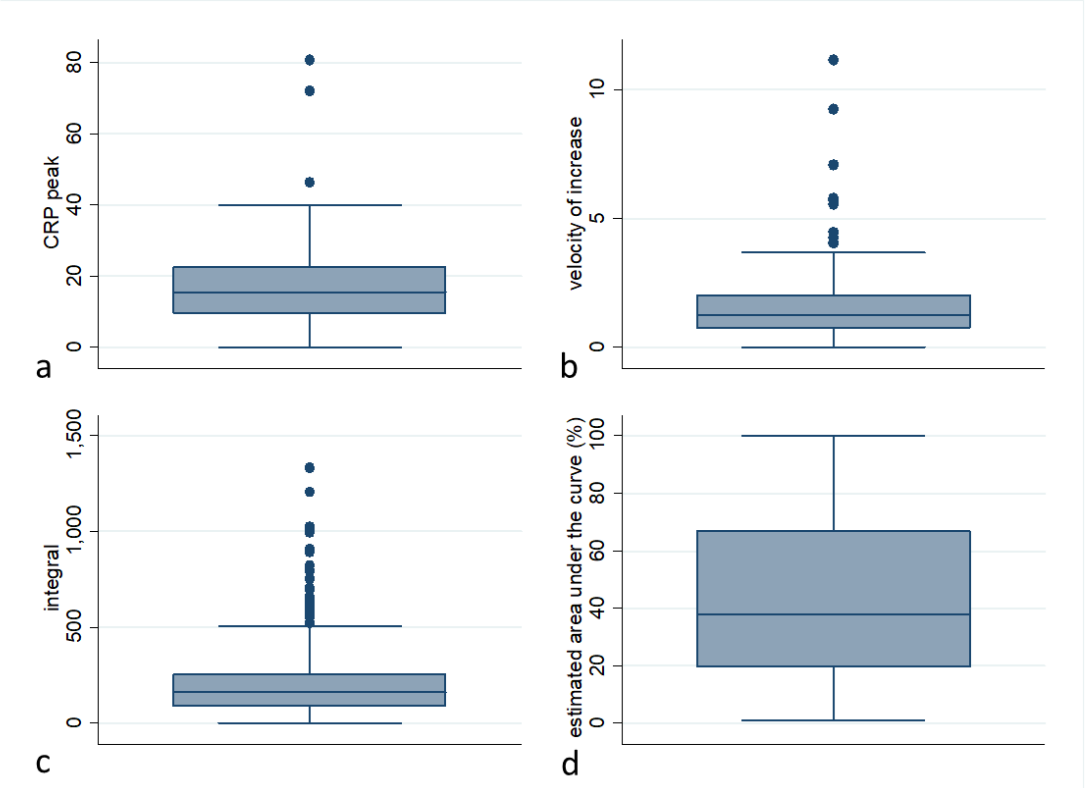

A

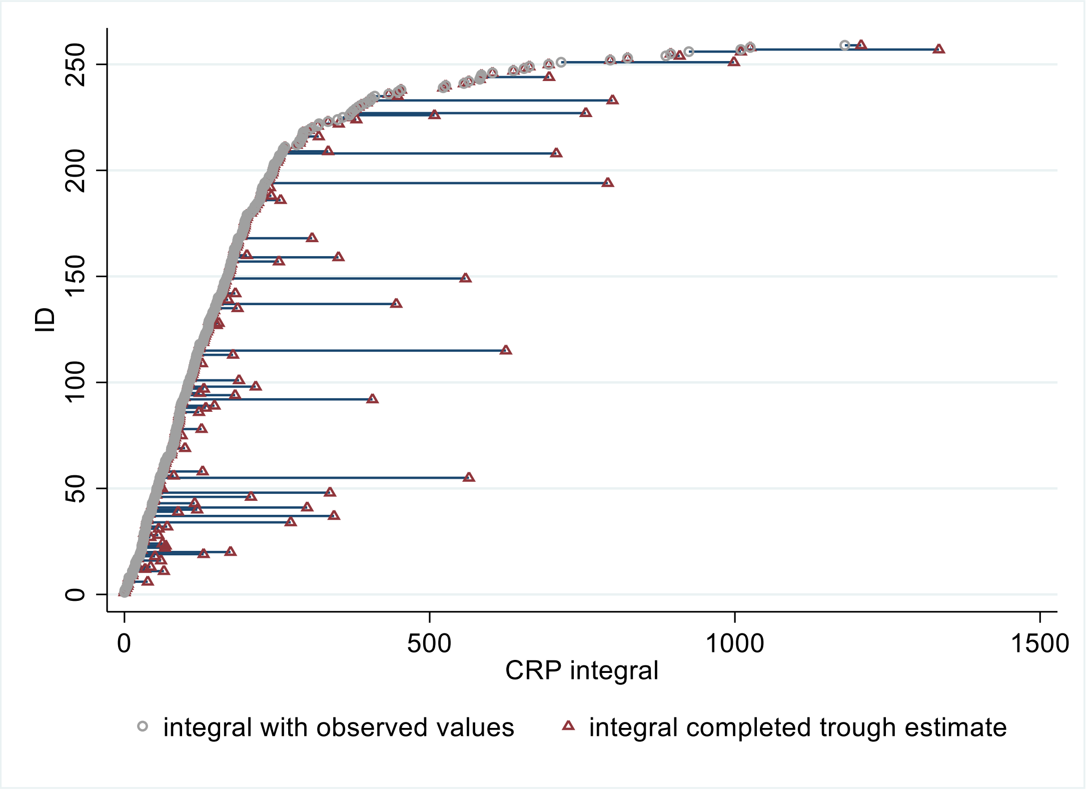

B

Supplementary Figure 2: A) Distribution of CRP descriptors (peak, a; velocity of increase, b; and integral, c) and percentage of the curve which was estimated by means of imputation (d). The right tail of the curve was not estimated in 186 (71.8%) patients. In fact, a complete CRP curve (ending value <1 mg/dl) was available in 130 patients, and 56 other patients had an ending value between 1 and 3 mg/dl, which was approximated to 0. B) integral values with and without adding the right tail.

Supplementary Table 2. Spearman' correlation between the three CRP curve descriptors

	**Velocity**	**Peak**	**Integral**
**Velocity**	1		
**Peak**	0.7855	1	
**Integral**	0.5384	0.8244	1

Supplementary Figure 3

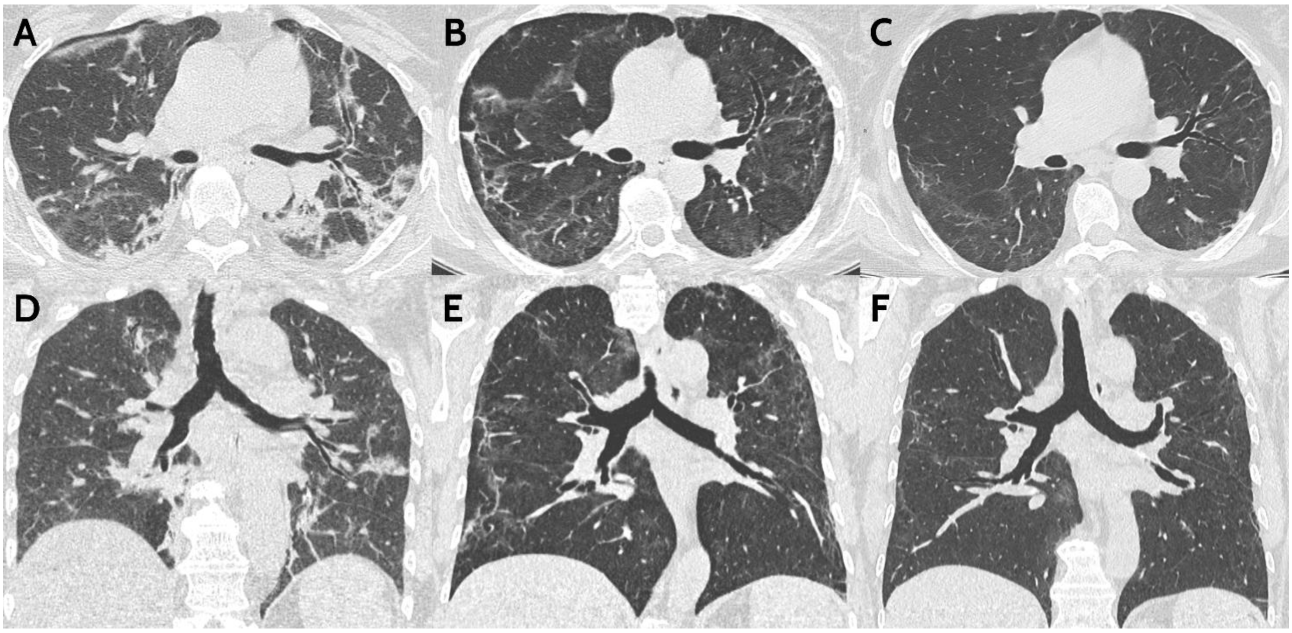

Supplementary Figure 3: axial (A, B, C) and coronal (D, E, F) CT images showing bilateral consolidations with predominant peripheral distribution at baseline (A, D) and residual abnormalities at 2-3 months (B, E) and 6-7 months (C, F) characterized by subtle ground-glass opacities and peripheral bands becoming progressively less apparent, suggestive of OP sequelae.

Supplementary Table 3. Logistic models adjusted for age and sex, including different combinations of CRP curve descriptors (peak, velocity, integral) to predict persistent CT lung abnormalities at 2-3 and 6-7 months.

	**CT lung abnormalities at 2-3 months**				**CT lung abnormalities at 6-7 months**			
	N=259				*N=241*			
	OR (95% CI)	*R^2*	*AIC*	*AUROC*	*OR (95% CI)*	*R^2*	*AIC*	*AUROC*
		0.138	243.2	0.752		0.120	296.0	0.723
Peak std	1.79 (1.23-2.62)				1.72 (1.27-2.34)			
Age	1.07 (1.04-1.10)				1.07 (1.04-1.10)			
Sex Male	1				1			
Female	0.80 (0.40-1.59)				0.90 (0.49-1.66)			
		0.109	251.3	0.726		0.103	301.4	0.710
Velocity std	1.29 (0.94-1.77)				1.68 (1.16-2.42)			
Age	1.07 (1.04-1.10)				1.06 (1.04-1.09)			
Sex Male	1				1			
Female	0.82 (0.41-1.64)				0.93 (0.51-1.70)			
		0.134	244.4	0.757		0.141	289.0	0.748
Integral std	1.64 (1.18-2.29)				2.24 (1.53-3.28)			
Age	1.07 (1.04-1.10)				1.06 (1.03-1.09)			
Sex Male	1				1			
Female	0.85 (0.42-1.71)				0.94 (0.51-1.75)			
		0.140	244.7	0.751		0.121	297.7	0.722
Peak std	2.01 (1.21-3.33)				1.60 (1.07-2.38)			
Velocity std	0.87 (0.59-1.29)				1.14 (0.72-1.81)			
Age	1.07 (1.04-1.11)				1.07 (1.04-1.10)			
Sex Male	1				1			
Female	0.76 (0.38-1.54)				0.91 (0.50-1.68)			
		0.142	244.3	0.759		0.142	290.8	0.746
Peak std	1.46 (0.84-2.53)				1.11 (0.73-1.69)			
Integral std	1.26 (0.78-2.04)				2.02 (1.18-3.47)			
Age	1.07 (1.04-1.10)				1.06(1.04-1.09)			
Sex Male	1				1			
Female	0.83 (0.41-1.68)				0.94(0.50-1.74)			
		0.137	245.6	0.756		0.141	290.9	0.747
Velocity std	0.81 (0.51-1.29)				1.11 (0.70-1.75)			
Integral std	1.90 (1.19-3.04)				2.13 (1.38-3.31)			
Age	1.07 (1.04-1.10)				1.6 (1.03-1.09)			
Sex Male	1				1			
Female	0.82 (0.41-1.67)				0.94 (0.51-1.75)			

The following variables were included for building the models with a backward strategy: CRP peak, CRP integral, CRP velocity, age, sex. Smoke, asthma, and COPD were also tested but did not improve the models’ performance. Pseudo R^2, Akaike information criterion (AIC), and area under ROC curve (AUROC) are reported for each model. For standardized variables we report OR for one standard deviation increase of the variable; for age we report the OR for one year increase.

Supplementary Table 4. Sensitivity analyses reporting the models previously selected in the whole population and after excluding patients with CT post-ventilatory damage (n=7) and active smokers (n=4).

	CT lung abnormalities at 2-3 months				CT lung abnormalities at 6-7 months			
	N	*OR (95% CI)*	*p*	*AUC*	*N*	*OR (95% CI)*	*p*	*AUC*
Whole population								
	259			0.752	241			0.748
Peak std		1.79 (1.23-2.62)	0.003					
Integral std						2.24 (1.53-3.28)	<0.001	
Age		1.07 (1.04-1.10)	<0.001			1.06 (1.03-1.09)	<0.001	
Sex M		1				1		
F		0.80 (0.40-1.59)	0.52			0.94 (0.51-1.75)	0.84	
Excluding patients with CT post-ventilatory changes								
	252			0.747	234			0.740
Peak std		1.74 (1.19-2.55)	0.004					
Integral std						2.10 (1.42-3.09)	<0.001	
Age		1.07 (1.04-1.10)	<0.001			1.06 (1.03-1.09)	<0.001	
Sex M		1				1		
F		0.79 (0.40-1.59)	0.52			0.92 (0.49-1.72)	0.79	
Excluding active smokers								
	255			0.765	237			0.755
Peak std		1.88 (1.27-2.78)	0.002					
Integral std						2.33 (1.57-3.46)	<0.001	
Age		1.08 (1.05-1.11)	<0.001			1.07 (1.04-1.10)	<0.001	
Sex M		1				1		
F		0.79 (0.39-1.59)	0.50			0.91 (0.49-1.71)	0.78	

OR, odds ratio; CI, confidence interval; AUC, area under the ROC curve.

Supplementary Table 5. Linear regression models for CT parenchymal extension at 2-3 and at 6-7 months.

	CT parenchymal extension at 2-3 months			CT parenchymal extension at 6-7 months		
	**N**	**Beta (95% CI)**	**P**	**N**	**Beta (95% CI)**	**P**
**Model**	258			218		
CRP peak std		3.67 (1.54;5.81)	0.001		-	
CRP integral std		-			3.06 (0.86;5.25)	0.007
Age		0.42 (0.24;0.60)	0.000		0.27 (0.09;0.44)	0.004
Sex M		1	0.472		1	
F		-1.70 (-6.34;2.94)			-1.40 (-5.65;2.84)	0.516
**Adjusted model**	258			218		
CRP peak std		3.16 (1.02;5.30)	0.004		-	
CRP integral std		-			2.85 (0.65;5.05)	0.011
Baseline parenchymal extension <20%		1			1	
20-39%		7.80 (-1.07;16.67)	0.084		2.42 (-6.64;11.48)	0.599
40-59%		2.62 (-5.63;10.87)	0.532		-1.43 (-9.89;7.03)	0.739
≥60%		9.65 (0.37;18.93)	0.042		3.24 (-5.94;12.41)	0.488
Age		0.43 (0.24;0.61)	0.000		0.29 (0.11;0.47)	0.002
Sex M		1	0.444		1	0.444
F		-1.80 (-6.43;2.83)			-1.67 (-5.97;2.62	

CI, confidence interval; CRP, C-reactive protein.
